# Supplementary material for: Association between oral microbiome and seven types of cancers in East Asian population: a two-sample Mendelian randomization analysis
Source: Front Mol Biosci. 2023 Nov 21;10:1327893. doi: 10.3389/fmolb.2023.1327893 (PMC10702768; doi:10.3389/fmolb.2023.1327893)
Supplement: Supplementary file 1 [file DataSheet1.ZIP › Supplementary Materials/MR plots for tongue/tongue═╝/Prostate cancer/pheno.3478_to_prostate cancer_leave_one_out.pdf]

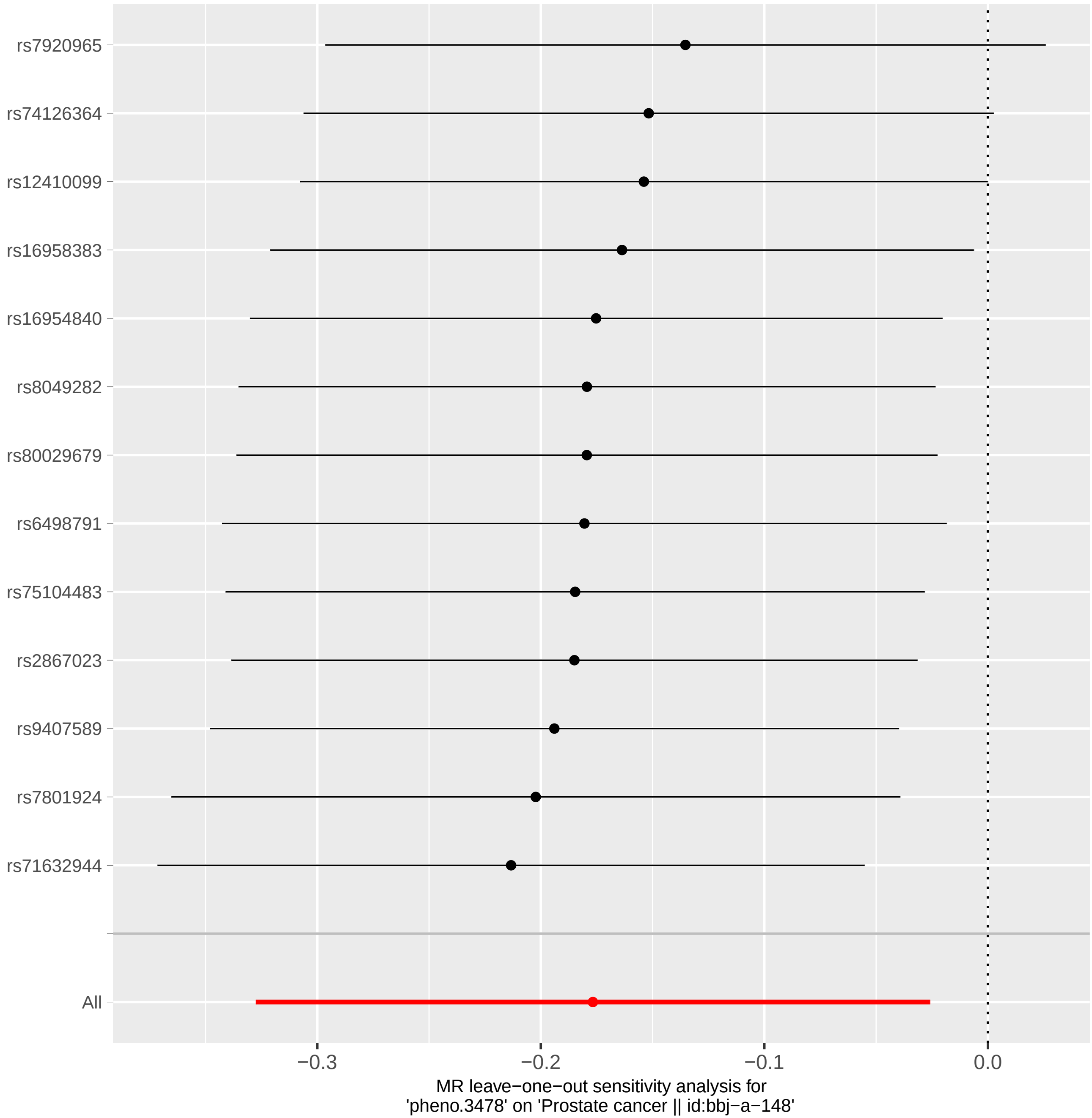
rs7920965
rs74126364
rs12410099
rs16958383
rs16954840
rs8049282
rs80029679
rs6498791
rs75104483
rs2867023
rs9407589
rs7801924
rs71632944
All
−0.3
−0.2
−0.1
0.0
MR leave−one−out sensitivity analysis for
'pheno.3478' on 'Prostate cancer || id:bbj−a−148'
